# Randomized Comparison of Two Vaginal Self-Sampling Methods for Human Papillomavirus Detection: Dry Swab versus FTA Cartridge

**DOI:** 10.1371/journal.pone.0143644

**Published:** 2015-12-02

**Authors:** Rosa Catarino, Pierre Vassilakos, Aline Bilancioni, Mathieu Vanden Eynde, Ulrike Meyer-Hamme, Pierre-Alain Menoud, Frédéric Guerry, Patrick Petignat

**Affiliations:** 1 Division of Gynecology, Department of Gynecology and Obstetrics, Geneva University Hospitals, Geneva, Switzerland; 2 Faculty of Medicine, University of Geneva, Geneva, Switzerland; 3 Geneva Foundation for Medical Education and Research, Geneva, Switzerland; 4 Unilabs SA, Lausanne, Switzerland; University of Louisville School of Medicine, UNITED STATES

## Abstract

**Background:**

Human papillomavirus (HPV) self-sampling (self-HPV) is valuable in cervical cancer screening. HPV testing is usually performed on physician-collected cervical smears stored in liquid-based medium. Dry filters and swabs are an alternative. We evaluated the adequacy of self-HPV using two dry storage and transport devices, the FTA cartridge and swab.

**Methods:**

A total of 130 women performed two consecutive self-HPV samples. Randomization determined which of the two tests was performed first: self-HPV using dry swabs (s-DRY) or vaginal specimen collection using a cytobrush applied to an FTA cartridge (s-FTA). After self-HPV, a physician collected a cervical sample using liquid-based medium (Dr-WET). HPV types were identified by real-time PCR. Agreement between collection methods was measured using the kappa statistic.

**Results:**

HPV prevalence for high-risk types was 62.3% (95%CI: 53.7–70.2) detected by s-DRY, 56.2% (95%CI: 47.6–64.4) by Dr-WET, and 54.6% (95%CI: 46.1–62.9) by s-FTA. There was overall agreement of 70.8% between s-FTA and s-DRY samples (kappa = 0.34), and of 82.3% between self-HPV and Dr-WET samples (kappa = 0.56). Detection sensitivities for low-grade squamous intraepithelial lesion or worse (LSIL+) were: 64.0% (95%CI: 44.5–79.8) for s-FTA, 84.6% (95%CI: 66.5–93.9) for s-DRY, and 76.9% (95%CI: 58.0–89.0) for Dr-WET. The preferred self-collection method among patients was s-DRY (40.8% vs. 15.4%). Regarding costs, FTA card was five times more expensive than the swab (~5 US dollars (USD)/per card vs. ~1 USD/per swab).

**Conclusion:**

Self-HPV using dry swabs is sensitive for detecting LSIL+ and less expensive than s-FTA.

**Trial Registration:**

International Standard Randomized Controlled Trial Number (ISRCTN): 43310942

## Introduction

The burden of cervical cancer (CC) remains very significant, especially in developing countries, and accounts for around a quarter of a million deaths annually worldwide [1]. In the developed world, screening programs have shown a reduction in CC cases. On the contrary, in low-resource settings, cytology-based screening programs are difficult to implement.

Human papillomavirus (HPV) testing appears to overcome some inherent barriers of cytological screening. Detection sensitivity for cervical intraepithelial neoplasia grade 2 or worse (CIN 2+) of HPV testing is twice that of cytology [2–4]. Furthermore, screening via self-collected samples improves access to healthcare, reduces time and costs, and contributes to increased screening attendance [5, 6]. Self-sampling has also been proven as reliable as physician-obtained cervical samples for HPV and CIN 2+ detection [7–10]. Moreover, screening with a single round of HPV testing was associated with a significant decrease in CC mortality in low-resource countries [11, 12].

A great variety of collection devices has been used in studies on self-sampling for HPV-testing (self-HPV). The most common devices are tampons, swabs, cervicovaginal brushes, and cervicovaginal lavage. In current practice, samples for HPV testing are stored in a liquid-based medium, which requires careful handling owing to its flammability and toxicity. The need for stable transport and storage temperatures makes such testing methods difficult and costly to provide in developing countries. Furthermore, despite women’s high acceptance of self-HPV, they remain concerned about the validity of the method and are afraid of spilling the transport medium during the sampling procedure and transport [7, 13–15].

Dry storage and transport might be a valuable option. The FTA elute cartridge (Whatman Inc., Clifton, NJ, USA) is a dry carrier that immobilizes and stabilizes nucleic acids from fresh samples. This biohazard-free paper is chemically treated with proprietary reagents that lyse cells upon contact, denaturing proteins. The FTA cartridge contains an indicating dye that changes color when a sample is applied, thereby confirming that the procedure has been performed properly. Moreover, easy storage and transport at room temperature is possible.

Evidence shows good agreement for detection of HPV DNA between samples collected in the FTA cartridge (s-FTA) and in a liquid-based medium [13, 15–19]. Despite its advantages, the FTA cartridge is inconvenient in that DNA from the cytobrush used for specimen collection can be only partly transferred to the cartridge.

Alternatively, vaginal dry swabs (s-DRY) are inexpensive and are not usually associated with a great loss of cellular material for analysis. Studies have shown that self-HPV swabs can be successfully transported in a dry state at ambient temperature without compromising specimen integrity, and that there is good agreement (70–90%) for HPV detection between dry and wet swabs [20, 21].

Although the feasibility of both the FTA cartridge and s-DRY as self-collection methods for HPV detection has been compared in several studies to standard swab collection in a liquid medium, to our knowledge, the relationship between performance of the FTA cartridge and s-DRY for HPV detection has never been addressed. Our goal was to evaluate the acceptability and analytic performance of two dry storage and transportation devices, s-FTA and s-DRY, and comparing them with the current standard of HPV testing using physician-collected samples and a specimen transport medium (Dr-WET).

## Material and Methods

### Study population

From March 2014 through February 2015, we enrolled women from the colposcopy clinic of Geneva University Hospitals, Switzerland. Women were eligible if they were over 30 years old, and if they understood the study procedures and voluntarily agreed to participate by signing a written informed consent form. No follow-up was done. Pregnant women, those with a history of hysterectomy, and who did not consent to participate, were excluded. A sample of 150 women was consecutively recruited (20 women were excluded as they did not meet the inclusion criteria or declined to participate). The Cantonal Human Research Ethics Commission of Geneva (CCER) approved the study (February 17, 2014; CER: 14–011).

### Study procedure

All eligible women were invited to collect two self-samples (s-FTA and s-DRY). A physician subsequently collected a third cervical sample with a swab immersed in a collection medium (ESwab^™^; COPAN Italia, Brescia, Italy) for HPV testing (Dr-WET). The sequence of the two self-HPV tests was randomized to avoid potential bias that might favor the “first” test. A research nurse gave oral instructions to participants, who were instructed to wash their hands before the specimen collection procedure. Each participant received a package containing a specimen collection kit. The Rovers^®^ Viba-Brush^®^ (Rovers Medical Devices B.V., Oss, The Netherlands) was used for self-collection with the FTA cartridge, and the mid-turbinate flocked vaginal swab (FLOQSwabs^™^; COPAN Italia) used for self-collection with the s-DRY method. Recommendations were to hold the brush or swab by the end of its handle and insert the brush or swab into the vagina, avoiding contact with the external genitalia, until resistance was felt (at least 6 cm). While gently maintaining pressure, participants were instructed to turn the brush or swab three to five times. Subsequently, the brush was to be applied to the FTA cartridge by pressing it onto the middle of the indicated sample area and then rotate the brush three to five times across that area. The FTA elute matrix contains an indicating dye that changes color from purple to white when a sample has been applied correctly. Three small circles of approximately 3.0 mm diameter were cut from the center section of the FTA card with a disposable sterile carbon steel surgical blade, and placed into a 1.5-ml microcentrifuge tube. A new sterile blade was used for each card.

On the other hand, the flocked swab (s-DRY) was simply inserted into its plastic sleeve for storage and transport. During the subsequent colposcopy consultation, a physician also collected a sample for HPV testing.

After all specimen collection, participants completed a self-administered questionnaire to collect sociodemographic data and query acceptance of the test. The FTA-card punches, flocked swabs, and ESwabs were immediately forwarded to the laboratory for analysis.

This trial was registered at ISRCTN Registry as ISRCTN43310942 (for logistic reasons the trial was registered after the recruitment began; the authors confirm that all ongoing and related trials for this intervention are registered). The study protocol is available in S2 Text.

### DNA isolation and extraction

**Elution from FTA card**: The 3.0-mm FTA punch discs were washed in 500 μl of sterile water and pulse vortexed three times for 5 s. The water was removed from each tube and the punch discs were transferred to a new microfuge tube containing 200 μl distilled water, which was then transferred to a heating block at 95°C for 30 min. The sample was then removed, pulse vortexed three times for 5 s, and centrifuged for 30 s to separate the matrix from the eluate. The eluted material was transferred to a new tube and stored at –20°C until DNA extraction.

**Material recovery from dry swab sample (s-DRY)**: Each sample was placed into 1 ml of sterile phosphate-buffered saline (PBS), and each tube was pulse vortexed three times for 15 s before removing and discarding the swab collection device. Samples were then stored at −20°C until extraction.

**Material recovery from ESwab sample (Dr-WET)**: Tubes containing the Dr-WET sample were also pulse vortexed three times for 15 s, and then the swab was discarded. Samples were then stored at −20°C until automated extraction.

**DNA extraction**: The entire volume of recovered material was transferred into appropriate tubes and the volume adjusted to 600 μl with PBS. DNA was then extracted on an *m*2000*sp* instrument (Abbott Molecular, Des Plaines, IL, USA), according to manufacturer instructions. The DNA elution volume was 100 μl/sample.

**HPV and genotyping**: HPV analyses were performed using the Anyplex II HPV28 (H28) Detection test (Seegene, Seoul, South Korea). The H28 is a semi-quantitative real-time multiplex PCR 30 assay for screening and genotyping 28 HPV genotypes [22]. This test uses Dual-Priming Oligonucleotides (DPO^™^) and Tagging Oligonucleotide Cleavage and Extension (TOCE^™^) technologies, allowing simultaneous detection of 19 high-risk HPVs (including types 16, 18, 26, 31, 33, 35, 39, 45, 51, 52, 53, 56, 58, 59, 66, 68, 69, 73 and 82) and nine low-risk HPVs. The 19 high-risk HPVs detected in the test include HPV types defined by the *International Agency for Research on Cancer* as Group 1 (carcinogenic to humans), Group 2A (probably carcinogenic to humans), and Group 2B (possibly carcinogenic to humans). Analysis was done as recommended by the manufacturer.

### Statistical analysis

A sample size of 130 women was needed to validate differences in test performance of 10% or more, assuming a 40–50% prevalence of HPV infection in the selected population. Agreement between the collection methods according to cytological results was measured using the kappa statistic (ĸ) and corresponding standard deviation (SD). The proportion of positive agreement (PPA) between paired s-FTA and s-DRY samples was calculated using 2a/(f1 + g1), where a is the number of samples that were positive for HPV in both dry samples, f1 is the number of samples that were positive for s-FTA, and g1 is the number of samples that were positive for s-DRY. The same was done for self-collection methods (combined results of s-FTA and s-DRY) and Dr-WET.

Sensitivity and specificity for detection of high-risk HPV using Dr-WET as the gold standard was reported, as well as sensitivity and specificity of the three sampling methods for abnormal Pap smear. Because of the small number of high-grade squamous intraepithelial lesions (HSIL) or carcinomas in our population, we assessed sensitivity and specificity for low-grade squamous intraepithelial lesion or worse (LSIL+). The two-tailed McNemar’s test was used for mutual comparison of sensitivity and specificity. Positive and negative predictive values were also calculated. Data were analyzed using Stata Statistical Software Release 13 (StataCorp LP, College Station, TX, USA).

## Results

### Sample characteristics

**Fig 1:**
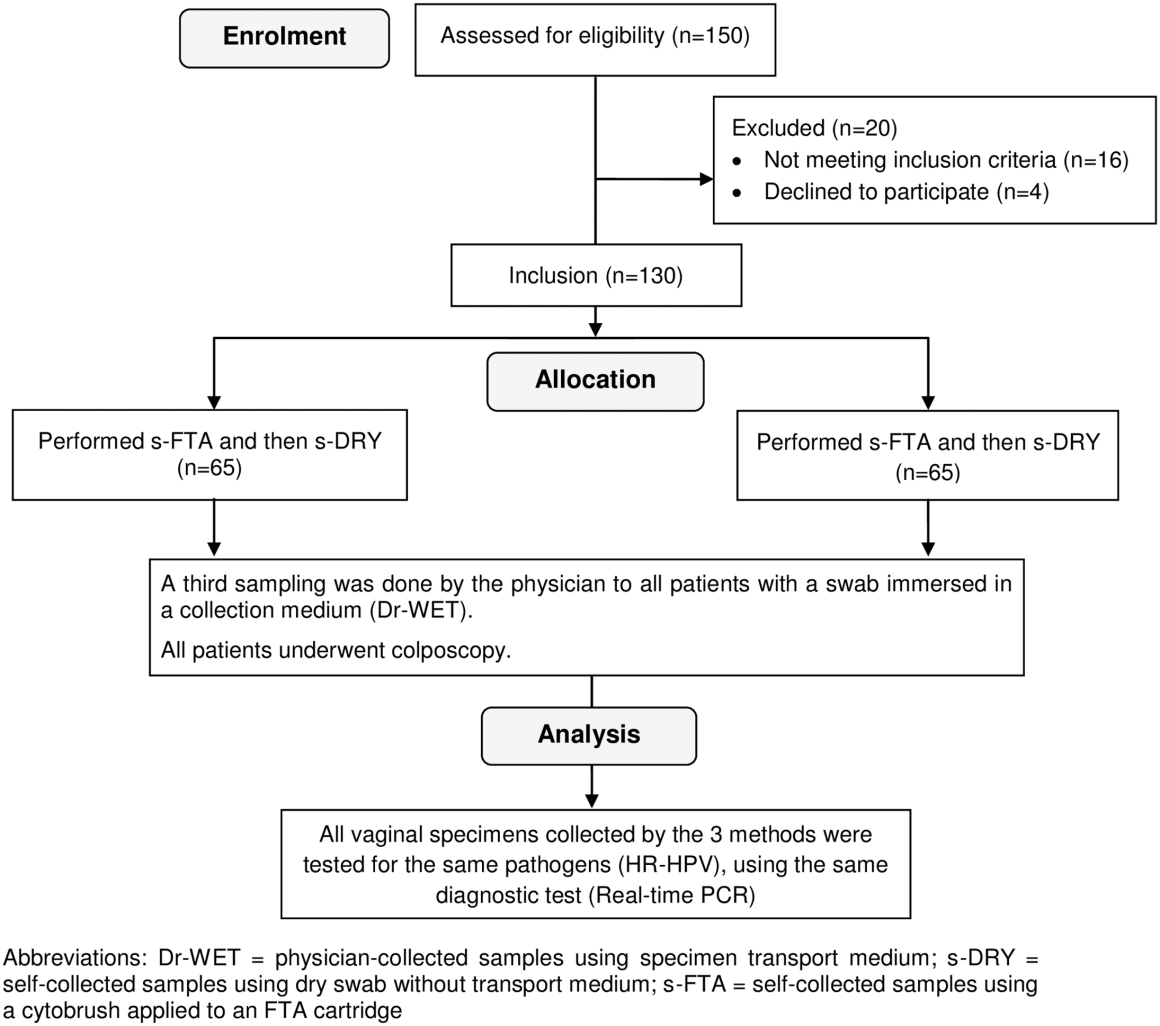
Flowchart of study participants.

One hundred and thirty patients were included in the study. Study flowchart is described in Fig 1. The median age of participants was 42 years [interquartile range (IQR) was 34–50], and the majority had a partner (77.7%). Sociodemographic data are represented in Table 1. The median delay between sampling and laboratory processing was 44 (IQR = 26–60) days.

**Table 1:** Sample sociodemographic characteristics (n = 130).

Variable	N. (%)
Age, median (IQR), y	42 (34–50)
Age groups, y	
30–39	50 (38.5)
40–49	47 (36.1)
≥50	33 (25.4)
Marital Status	
Without a partner	29 (22.3)
With a partner	101 (77.7)
Education	
Unschooled	2 (1.7)
Primary education	23 (19.7)
Secondary education	47 (40.2)
Tertiary education	45 (38.4)
Number of child (mean ± SD)	1.3±1.1

Abbreviations: IQR = Interquartile range N. = number; SD = standard deviation; y = years.

### Overall HPV detection with the three collection methods and HPV genotype distribution

HPV prevalence for high-risk types was 62.3% (95% CI: 53.7–70.2) detected by s-DRY, 56.2% (95% CI: 47.6–64.4) by Dr-WET, and 54.6% (95% CI: 46.1–62.9) by s-FTA. Infection with HPV types 16 and 18 alone was identified in 4.6% of participants, and infection together with other high-risk types in 13.8% of cases. Infection with high-risk genotypes other than types 16 and 18 was found in 50% of women.

The distribution of HPV genotypes is shown in Table 2 for high-risk types and in Table 3 for low-risk types. In general, a larger number of positive samples for each genotype were detected more often in s-DRY samples compared with the other collection methods.

**Table 2:** Number of high-risk human papillomavirus (HPV) genotypes* detected by each sample collection method.

hr-HPV	16	18	31	33	35	39	45	51	52	53	56	58	59	66	68	69	73	82
FTA	18	2	10	18	2	4	3	3	6	5	4	4	1	7	6	2	7	3
v-DRY	20	4	11	24	5	4	5	5	13	14	6	7	3	16	10	2	8	6
dr-WET	18	3	10	14	2	2	5	5	11	7	5	5	3	11	7	1	6	4

Abbreviations: Dr-WET = physician-collected samples using specimen transport medium; s-DRY = self-collected samples using dry swab without transport medium; s-FTA = self-collected samples using a cytobrush applied to an FTA cartridge; hr-HPV = high-risk human papillomavirus.

**Table 3:** Number of low-risk HPV genotypes* detected by each sample collection method.

lr-HPV	6	11	40	42	43	44	54	61	64	70	78
FTA	3	1	1	11	7	6	3	4	1	5	0
S-DRY	7	2	2	16	4	13	11	8	0	7	0
Physician	3	2	1	11	10	6	5	7	0	6	1

Abbreviations: Dr-WET = physician-collected samples using specimen transport medium; s-DRY = self-collected samples using dry swab without transport medium; s-FTA = self-collected samples using a cytobrush applied to an FTA cartridge; lr-HPV = Low risk human papillomavirus.

### Agreement between collection methods according to cytological results

Cytological results obtained in the 12 months prior to the patient recruitment date or during the study were considered in the data analysis (Tables 4 and 5). We found cytological diagnoses for 119 of the 130 patients enrolled. There were 61 normal diagnoses, 1 squamous cell carcinoma, 5 HSIL, 20 LSIL, 9 atypical squamous cells-cannot rule out high grade squamous intraepithelial lesion (ASC-H), 3 atypical glandular cells (AGC) and 20 atypical squamous cells of undetermined significance (ASC-US). Overall agreement between s-FTA and s-DRY samples when LSIL+ was present was 69.2% (ĸ = 0.27), and the PPA was 78.9%.

**Table 4:** Agreement and HPV positivity for each collection method, overall (n = 130) and according to cytology results (n = 119).

Cytology analysis	N. of Samples—Self-HPV					% Positive		Agreement (%)		Kappa (95% CI)
	Total	s-FTA+/s-DRY+	s-FTA+/ s-DRY-	s-FTA/ s-DRY+	s-FTA-/ s-DRY-	s-FTA cartridge % (95% CI)	s-DRY % (95% CI)	Overall	PPA	
Overall	130	70	9	29	22	60.8 (52.2–68.7)	76.2 (68.1–82.7)	70.8	78.7	0.34 (0.18–0.50)
NILM	61 (51.3)	26	2	17	15	45.9 (34.0–58.3)	72.1 (59.8–81.9)	68.3	72.2	0.38 (0.18–0.59)
ASC-US	20 (16.8)	14	4	0	2	90.0 (68.7–98.4)	70.0 (47.9–85.7)	80.0	87.5	0.41 (-0.01–0.83)
AGC	3 (2.5)	1	1	0	1	66.7 (20.2–94.4)	33.3 (56.3–79.8)	66.7	66.7	0.40 (-0.37–1.0)
ASC-H	9 (7.6)	7	1	1	0	88.9 (54.3–99.9)	88.9 (54.3–99.9)	77.8	87.5	-0.13 (-0.30–0.05)
LSIL	20 (16.8)	13	1	3	3	70.0 (47.9–85.7)	80.0 (57.8–92.5)	80.0	86.7	0.47 (-0.04–0.90)
HSIL	5 (4.2)	2	0	3	0	40.0 (11.6–77.1)	100 (51.1–100)	40.0	57.1	0
CC	1 (0.8)	0	0	1	0	0 (0–83.3)	100 (16.8–100)	0	0	0
LSIL+	26 (21.8)	15	1	7	3	61.5 (42.5–77.6)	84.6 (66.5–93.9)	69.2	78.9	0.27 (-0.07–0.61)

Abbreviations: n = number; NILM = Negative for intraepithelial lesion or malignancy; ASC-US = Atypical squamous cells of undetermined significance; ASC-H = Atypical squamous cells-cannot rule out high grade; AGC = Atypical glandular cell; LSIL = Low-grade squamous intraepithelial lesion; LSIL+ = Low-grade squamous intraepithelial lesion or worse; HSIL = High-grade squamous intraepithelial lesion; CC = Cervical Cancer; CI = confidence interval; PPA = Proportion of Positive Agreement; s-DRY = self-collected samples using dry swab without transport medium; s-FTA = self-collected samples using a cytobrush applied to an FTA cartridge.

**Table 5:** Agreement and HPV positivity for each collection method, overall (n = 130) and according to cytology results (n = 119).

Cytology analysis	N. of Samples—Self-HPV* vs. Dr-WET					% Positive		Agreement (%)		Kappa (95% CI)
	Total	Self+/ Dr-WET+	Self+/ Dr-WET -	Self-/ Dr-WET +	Self-/ Dr-WET -	Self-collection % (95% CI)	Dr-WET % (95% CI)	Overall	PPA	
Overall	130	85	23	0	22	83.1 (75.7–88.6)	65.4 (56.9–73.0)	82.3	88.1	0.56 (0.41–0.70)
NILM	61 (51.3)	34	12	0	15	75.4 (63.2–84.6)	55.7 (43.3–67.5)	80.3	85.0	0.58 (0.39–078)
ASC-US	20 (16.8)	15	3	0	2	90.0 (68.7–98.4)	75.0 (52.8–89.2)	85.0	90.9	0.50 (0.05–0.95)
AGC	3 (2.5)	1	1	0	1	66.7 (20.2–94.4)	33.3 (56.3–79.8)	66.7	66.7	0.40 (-0.37–1)
ASC-H	9 (7.6)	8	1	0	0	100 (65.5–100)	88.9 (54.3–99.9)	88.9	94.1	0
LSIL	20 (16.8)	15	2	0	3	85.0 (63.1–95.6)	75.0 (52.8–89.2)	90.0	93.8	0.69 (0.31–1)
HSIL	5 (4.2)	5	0	0	0	100 (51.1–100)	100 (51.1–100)	100	100	1
CC	1 (0.8)	0	1	0	0	100 (16.8–100)	0 (0–83.3)	0	0	0
LSIL+	26 (21.8)	20	3	0	3	88.5 (71.86.0)	76.9 (56.0–89.0)	89.7	93.0	0.61 (0.23–1)

Abbreviations: Dr-WET = physician-collected samples using specimen transport medium; n = number; NILM = Negative for intraepithelial lesion or malignancy; ASC-US = Atypical squamous cells of undetermined significance; ASC-H = Atypical squamous cells-cannot rule out high grade; AGC = Atypical glandular cell; LSIL = Low-grade squamous intraepithelial lesion; LSIL+ = Low-grade squamous intraepithelial lesion or worse; HSIL = High-grade squamous intraepithelial lesion; CC = Cervical cancer; CI = confidence interval; PPA = Proportion of Positive Agreement.

*Comprises results from Self-HPV using both collection methods (s-DRY and s-FTA).

Overall agreement between self-HPV and physician-collected samples when LSIL+ was present was 89.7% (ĸ = 0.61), and the PPA was 93.0%. Overall agreement between FTA cards and Dr-WET samples was 83.1%, and kappa was 0.64 (0.50–0.77). Overall agreement between s-DRY and Dr-WET samples was 85.2%, and the kappa was 0.64 (0.50–0.78)

### Number of oncogenic HPV genotypes detected

Agreement between the s-FTA versus Dr-WET, s-DRY versus Dr-WET, and s-FTA versus s-DRY for the number of oncogenic HPV types is shown in Table 6. Overall agreement between s-FTA versus Dr-WET was 70.8% with kappa = 0.56, between s-DRY versus Dr-WET was 72.3% with kappa = 0.61, and between s-FTA versus s-DRY was 54.6% with kappa = 0.35. Detection of three or more oncogenic HPV types was more common in s-DRY samples (17.7%) relative to s-FTA and Dr-WET samples (4.6% and 8.4%, respectively).

**Table 6:** Comparison between number of oncogenic HPV genotypes* detected in the three different samples (s-FTA vs. Dr-WET; s-FTA vs. s-DRY; s-DRY vs. Dr-WET).

	N. of hr-HPV types detected in Dr-WET samples					N. of hr-HPV types detected in s-DRY samples				
N. of hr-HPV types detected in s-FTA samples	0	1	2	3+	Total	0	1	2	3+	Total
0	48	7	3	0	58	38	15	2	3	58
1	9	29	9	4	51	9	20	13	9	51
2	0	5	9	1	15	0	3	7	5	15
3+	0	0	0	6	6	0	0	0	6	6
Total	57	1	21	11	130	47	38	22	23	130
N. of hr-HPV types detected in s-DRY samples										
0	43	3	1	0	47					
1	12	26	0	0	38					
2	1	5	15	1	22					
3+	1	7	5	10	23					
Total	57	41	21	11	130					

Abbreviations: n. = number; Dr-WET = physician-collected samples using specimen transport medium; s-DRY = self-collected samples using dry swab without transport medium; s-FTA = self-collected samples using a cytobrush applied to an FTA cartridge

*Oncogenic genotypes include types 16, 18, 26, 31, 33, 35, 39, 45, 51, 52, 53, 56, 58, 59, 66, 68, 69, 73, 82 (Seegene, Anyplex^™^ II HPV28)

### Clinical performance of self-collection and physician-collection methods

Clinical performance of self-collection and physician-collection methods using the Dr-WET sample results or the cytological results (LSIL+) as gold standard is represented in Table 7.

**Table 7:** Clinical performance of self-collection and physician-collection methods

Variables	s-FTA and s-DRY performances, using Dr-WET results as gold-standard			
	Sensitivity (95% CI)	Specificity (95% CI)	PPV (95% CI)	NPV (95% CI)
s-FTA	85.4 (76.4–91.5)	82.2 (68.7–90.7)	89.9 (81.3–94.8)	75.5 (61.9–85.4)
s-DRY	96.5 (90.1–98.8)	62.2 (47.6–74.9)	82.2 (74.2–89.0)	90.3 (75.1–96.7)
p value*	0.020	0.050	.	.
Variables	s-FTA, s-DRY and Dr-WET performances, using pathological cytological results as gold-standard (LSIL+)			
	Sensitivity (95% CI)	Specificity (95% CI)	PPV (95% CI)	NPV (95% CI)
s-FTA	64.0 (44.5–79.8)	39.1 (29.8–49.4)	22.2 (14.2–33.1)	80.0 (66.2–89.1)
s-DRY	84.6 (66.5–93.9)	28.0 (19.9–37.8)	24.7 (16.9–34.6)	86.7 (70.3–94.7)
p value*	0.059	0.008		
Dr-WET	76.9 (58.0–89.0)	37.6 (28.5–47.8)	25.6 (17.3–36.3)	85.4 (71.6–93.1)
p value(vs. FTA)	0.180	0.800	.	.
p value(vs. s-DRY)	0.160	0.020	.	.

Abbreviations: CI = confidence interval; PPV = Positive Predictive Value; NPV = Negative Predictive Value; Dr-WET = physician-collected samples using specimen transport medium; s-DRY = self-collected samples using dry swab without transport medium; s-FTA = self-collected samples using a cytobrush applied to an FTA cartridge

*The p-value was calculated with McNemar's test.

### Sample preferences and opinion about the collection methods

Sample preferences and opinion about the collection methods are represented in Table 8. The preferred self-collection method among participants was s-DRY (40.8% vs. 15.4%), and a larger number of women reported feeling very confident using it (48.5%). One hundred and seventeen (90.0%) women affirmed that they were prepared to self-collect a vaginal sample at home. Half of the participants (50.0%) expressed a preference for HPV testing by self-collection at home rather than going to a clinic for a pelvic exam and cytology testing.

**Table 8:** Sample preferences and opinions regarding collection methods (n = 130).

Variable	s-FTA	s-DRY
	n. (%)	n. (%)
Which one of the two self-collection methods do you prefer?	20 (15.4)	53 (40.8)
Do you that both self-collection methods have the same reliability?		
I don't know	86 (66.7)	
Yes	24 (18.6)	
No	18 (14.0)	
If not, which one do you think is more effective?	14 (10.8)	6 (4.6)
Do you think that the self-collection was painful?		
Not painful at all	98 (75.4)	108 (83.1)
Slightly painful	25 (19.2)	16 (12.3)
Moderately to very painful	7 (5.4)	6 (4.6)
In a scale of 0–4, how painful was the procedure? (mean ± SD)	1.3±0.60	1.2±0.58
Are you confident that you have correctly performed the self-collection?		
Not sure at all/ Slightly confident	27 (20.8)	17 (13.1)
Moderately confident	49 (37.7)	49 (37.7)
Very confident	51 (39.2)	63 (48.5)
In a scale of 0–4, how confident are you about the procedure? (mean ± SD)	1.2±0.41	1.4±0.58
How do you classify the methods' complexity?		
Easy	91 (70.0)	111 (85.4)
Moderate to Complex	39 (30.0)	19 (14.6)
Which one would you recommend to your family and friends?	13 (10)	43 (33.1)
Would you be ready to do the sample self-collection at home using written instructions?		
No	13 (10.0	
Yes	117 (90.0)	
If yes, with which one of the methods?	8 (6.2)	32 (24.6)
In the future would you rather do self-collection for HPV testing or continue going to the doctor for a Pap test?		
Pap Test with doctor	63 (48.5)	
Self-Collection for HPV testing	63 (48.5)	

Abbreviations: n = number; SD = standard deviation.

## Discussion

To our knowledge, this is one of the first studies to compare acceptability and analytic performance between two dry storage and transportation devices for HPV detection, the FTA cartridge and dry swab.

Overall, detection of HPV was significantly less common with the s-FTA method relative to s-DRY or Dr-WET, using the same test for HPV detection (Anyplex II HPV28). This finding is consistent with a previous trial in Barcelona (16] but contradicts the findings of other studies [13, 15, 18, 23] in which s-FTA appeared to be a suitable dry transport carrier. However, in the former studies, the collection method or storage medium to which the s-FTA sample was compared differed from ours. In a study by Gonzalez et al. [18], there was increased HPV detection of any type in samples collected in an s-FTA medium than in those in a PreservCyt medium (54.5% versus 45.8%). The reduced detection of HPV in s-FTA samples in our study is most likely attributable to insufficient cellular material collected by the patient or inadequate transfer of material from the brush onto the card.

In our study, sensitivity was much lower for the s-FTA method than the s-DRY method with use of cytological results as the gold standard (64.0% vs. 84.6%), even when a real-time PCR method was used for HPV testing in both collection methods. Indeed, previous studies have demonstrated that s-FTA is a favorable method for HPV diagnosis in the context of GP5/6-PCR use, but not Hybrid Capture 2 [13, 18]. In a study by Geraets et al. [16], in which the FTA-based self-collection method was compared with physician-collected cervical samples stored in a liquid medium, a 98% sensitivity in CIN 2+ detection for the physician-collected sample using GP5+/6+ testing was found, which is comparable to the combination of self-collection with s-FTA and SPF10 HPV detection (sensitivity 95.9%). These results indicate that clinical performance of HPV detection is determined by both the sample collection method and test used.

Overall, several studies have shown substantial agreement for PCR HPV detection and genotyping between cervical cells collected using a liquid-based medium and specimens collected using FTA cartridges, among both self-collected and clinician-collected samples [13–15, 18, 23]. In Geraets et al. [16], overall agreement was 89.0% between HPV test results with the s-FTA method and corresponding physician-collected samples, giving a kappa of 0.73 (95% CI: 0.63–0.84). These results are consistent with ours. However, if we compare the s-FTA samples with the s-DRY method, agreement was much less than expected (70.8%; kappa = 0.34; 95% CI: 0.18–0.50).

This study has some limitations that must be addressed. The self-collected samples were always done before the physician-collected sample, creating a bias in favor of the self-HPV. Another limitation is the uncertainty that our findings can be generalized to other populations with low HPV prevalence; our study was based on adult women in a colposcopy clinic with a high prevalence of HPV infection.

The strengths of our study include the possibility for comparing the s-FTA, s-DRY and Dr-WET methods within categories of cytological diagnoses and, more specifically, for evaluating the performance of s-FTA and s-DRY among women with an LSIL+ diagnosis. Furthermore, by randomizing the sequence of the two self-collection methods, we did not favor one method over the other.

Although the FTA card may have some advantages such as the assurance of correct test performance by means of the color indicator, it is not as sensitive as other collection methods and has a higher cost. Indeed, by comparing the regular prices of the card (Whatman Indicating FTA^™^ Elute Micro Card) with the dry swab (COPAN FLOQSwabs^™^), we found that the card is five times more expensive (~5 USD per card vs. ~1 USD per swab). This does not even consider the price of the entire kit, which includes the brush for collection and puncher for extraction. In addition to these disadvantages, the method would be difficult to implement in a context of low human and material resources, owing to laborious sample processing. In the present study, testing was done according to strict standard laboratory procedures to avoid PCR contamination. Punching of the FTA card is not only expensive, it is labor-intensive, and cross-contamination can readily occur if rigorous techniques are not used. In this study, a disposable surgical blade was used and we have no reason to believe that this would affect card performance, as this technique has been previously used without compromising the results [17]. Furthermore, transferring a sample collected with a brush onto the surface of an FTA card might result in a nonrepresentative sample. Additionally, in cases of high-grade CIN (with greater HPV DNA integration), HPV copies per cell tend to be reduced and dysplastic cervical cells may be less likely to be transferred onto a solid substrate like FTA [14]. Since the sensitivity for detecting low- and high-grade cervical dysplasia using the FTA card is unsatisfactory, its use should be reconsidered in light of better existing alternatives.

In summary, the benefits of dry carriers are appealing, owing to accessibility and simplicity. The present study used a cost-effective strategy to promote use and validate the most optimal technique for HPV screening. Contrary to the optimistic results of other studies [14, 15, 17, 18, 23], we did not find the FTA method to be as promising for HPV testing. We found that the FTA cartridge is not only less sensitive than swabs but is also more expensive than other methods. In our view, the FTA method is inappropriate for use in low-resource settings and may only be slightly appealing for self-HPV testing in developed countries, because of a pleasing modern design that may help reassure women and motivate them to perform self-sampling at home. Based on the findings of this study, dry swabs should be adopted in future projects within low-resource settings, where it may be a great asset for CC screening.

## Supporting Information

S1 Protocol Study Protocol. (PDF)

S1 TREND Checklist TREND Checklist. (PDF)
