# Supplementary material for: Randomized Comparison of Two Vaginal Self-Sampling Methods for Human Papillomavirus Detection: Dry Swab versus FTA Cartridge
Source: PLoS One. 2015 Dec 2;10(12):e0143644. doi: 10.1371/journal.pone.0143644 (PMC4668032; doi:10.1371/journal.pone.0143644)
Supplement: S1 Protocol — (PDF) [file pone.0143644.s001.pdf]

Randomized comparison of two self-sampling methods (Vaginal dry swabs vs. FTA-elute cartridge) for HPV detection.

Study Protocol

March 2014

Version 2

CONFIDENTIAL

Main investigator:

Rosa Catarino, Gynecology service, HUG

RosaIsabel.PintoCatarino@hcuge.ch

Co-investigators:

Pr Patrick Petignat, Gynecology service and Surgical Gynecologic Oncology Unit, HUG

Patrick.Petignat@hcuge.ch

PD, Dr Pierre Vassilakos, Geneva Foundation for Medical Education and Research, Geneva.

pierrevassilakos@bluewin.ch

Pr Michel Boulvain, Obstetrics Service and Development Unit, HUG

Michel.Boulvain@hcuge.ch

Aline Bilancioni, referent nurse in colposcopy

Aline.Kindynis@hcuge.ch

Dr Martine JACOT-GUILLARMOD, Department of Gynecology & Obstetrics, CHUV

Martine.Jacot-Guillarmod@chuv.ch

Abstract

Background: Human papillomavirus (HPV) self-tests are likely to be used in cervical cancer screening. Most HPV testing is performed on cervical smears stored in liquid-based medium. Dry filters are an alternative.

Objective: Evaluate the acceptability and analytic performance of two dry storage and transportation devices, FTA elute cartridge and Vaginal Dry Swabs.

Material and method: Vaginal specimens for HPV self-tests will be collected with two different methods for each woman. Randomization will determine which one of the two tests will be performed primarily: a Self-HPV using dry swabs (v-DRY) or vaginal specimen applied in FTA cartridge (v-FTA). During consultation, the physician will collect a cervical sample using specimen transport medium (v-STM). HPV types will be identified by Real-time PCR. A sample of 130 patients will be included. Women will complete a self-administered questionnaire on demographics and preference for sampling method. Agreement between collection methods and HPV risk categories will be measured using the kappa statistic (κ).

Expected results: Evaluate the accuracy of two self-HPV methods as a strategy for HPV diagnosis.

Key words: self-sampling, FTA cartridge, dry swabs, wet swabs, cervical cancer, human papillomavirus (HPV)

CONFIDENTIAL

1. Background

There are about 500,000 new cases of cervical cancer per year, with 85% of them occurring in developing countries, making it the second most common cancer among women worldwide and the first one in Africa. Cervical cancer is the leading cause of cancer-related death between women living in low resource settings, accounting for 247,590 deaths per year (90% of global deaths) [1].

Screening programs have shown a reduction of cervical cancer cases in the developed world. The sensitivity in the detection of high-grade cervical intraepithelial neoplasia with the HPV testing is twice as great compared with cytology-based tests [2-4]. In addition, screening via self-samples improves the access to healthcare, reduce the costs and save time for patients and providers, increasing the screening attendance [5, 6]. This is particularly important in low resource countries, where a cytology-based screening program is difficult to put in practice because of the lack of material resources and of human skills needed to analyze the results. HPV testing may overcome some of these barriers. It has been shown that in these countries, screening with single round of HPV testing is associated with significant decrease in cervical cancer-related mortality [7, 8]. In addition, self-collected samples have proved to be as reliable as physician-obtained cervical samples for the detection of HPV [9-11].

A great variety of collection devices have been used in studies on HPV self-sampling. Most common devices were tampons, swabs, cervicovaginal brushes, and cervicovaginal lavage. In current practice, HPV testing is stored in liquid-based medium, which requires careful handling, due to its flammability and toxicity. The need for stable carrying and storage temperatures makes it difficult and costly to provide in developing countries. Furthermore, despite women's high acceptability regarding self-sampling for HPV testing, they are still concerned about the validity of the method and they are afraid of spilling out the transport medium during the sampling procedure and transport [9, 12-14].

Dry storage and transport might be a valuable option. In particular, the FTA elute cartridge (Whatman, Inc., Clifton, NJ) is a dry carrier that immobilizes and stabilizes nucleic acids from fresh samples applied. This biohazard free paper is chemically treated with proprietary reagents that lyse cells upon contact, denaturing proteins. The FTA contains an indicating dye that changes color where a sample is applied, thereby confirming that women performed the procedure properly. It allows easy storage and transportation at room temperature and the DNA extraction is very simple. Evidence shows a good agreement for HPV DNA detection between samples collected in FTA cartridge and liquid-based medium [14, 17-21]. Geraets et al. compared FTA-based self-collection method with physician-collected cervical samples stored in liquid-based and they found that the combination of physician-collected specimen and GP5+/6+ testing demonstrated a sensitivity of 98% and specificity of 48.1% for CIN2+ detection, comparable to a test system of FTA-based self-collection and SPF10 hrHPV detection (sensitivity 95.9% and specificity 42.9%). These results show that the clinical performance of hrHPV detection is determined by both the sample collection system and the test method [17]. In 2009, Lenselink et al. compared self-collected samples at home placed in both media from 51 women aged 18 to 29 years with an agreement of 100% [22].

Despite its advantages, the FTA cartridge has the inconvenience that the DNA from the brush can be only partly transferred to the cartridge.

CONFIDENTIAL

Alternatively, dry vaginal swabs (v-DRY) are less expensive and they usually are not associated with great loss of material for analysis. Studies have shown that Self-HPV swabs can be successfully transported in a dry state at ambient temperature without compromise specimen integrity and that there is a good agreement (70-90%) for HPV detection between dry and wet swabs [15, 16].

Although the feasibility of both FTA cartridge and vaginal dry swabs (v-DRY) as self collection methods for HPV detection has been compared to standard wet swabs in several studies, the relation between the performance of FTA cartridge and v-DRY for HPV detection as never been addressed to our knowledge. Our goal was to evaluate the feasibility of Self-HPV using dry swabs (v-DRY) compared with Self-HPV using FTA cartridge, comparing it to the actual standard, the HPV test on physician collected samples using specimen transport medium (v-STM).

In summary, the benefits of the dry carriers are appealing due to its accessibility and simplicity. The present study uses a cost-effective strategy that will contribute to the development and validation of these techniques for HPV screening.

2. Objective

Evaluate the acceptability and analytic performance of two dry storage and transportation devices, FTA elute cartridge and Vaginal Dry Swabs.

3. Material and method

Inclusion Criteria:

- =/> 30 years
- Attending colposcopy clinic
- Understands study procedures and accepts voluntarily to participate by signing the informed consent form (ICF)

Exclusion Criteria:

- Pregnancy
- Previous Hysterectomy
- Virgin
- Not able to comply with protocol study.

Study design:

Women will be invited to perform two self-sampling (FTA and v-DRY). Randomization will determine the sequence of the two tests, avoiding potential bias that may advantage the “first” test. All specimens will be tested for the same pathogens (HR-HPV) using the same diagnostic test (Real-time PCR).

CONFIDENTIAL

Study procedure:

A research nurse will give instructions to the patients and ICF will be obtained. For specimen collection, participants will be instructed to wash their hands before the procedure. Each participant will receive a package containing specimen collection kit. Recommendations will be to hold the brush by the end of the handle, to insert the brush into the vagina, avoiding contact with the external genitalia, until they meet resistance (at least 6 cm). Once they meet resistance, they shall gently turn the brush three to five times. Subsequently the brush shall be applied to the FTA cartridge by pressing it onto the sample area indicated and then rotate de swab 3-5 times, or just put in inside a plastic sleeve (v-DRY).

During colposcopy consultation, the physician will also collect a sample using specimen transport medium (v-STM) for HPV testing. We will look for the Pap test results of the participating women.

At the end, women will complete a self-administered questionnaire on demographics and preference for sampling method.

HPV analysis:

The HPV analysis will be performed by Real-time PCR. Delay between sampling and lab processing will be noted.

Statistical analysis:

- Agreement between collection methods in terms of HPV risk categories will be measured using kappa statistic (κ) with a precision of 10% (95% confidence interval). This measure agreement is 0 when the amount of agreement is what would be expected by chance and 1 when there is perfect agreement.

- Sensitivity and specificity to detect high-risk HPV using v-STM as gold standard will also be reported (table 2x2). Sensitivity and specificity of the three sampling methods for abnormal Pap smear or abnormal biopsy results will also be determined. Because of the small-expected number of high-grade squamous intraepithelial lesion or carcinomas in our population, we will assess sensitivity and specificity for low-grade squamous intraepithelial lesion or greater lesions.

- Positive and negative predictive values will also be calculated (positive and negative predictive values to detect high-risk HPV using the physician's collected samples as gold standard; positive and negative predictive values of the three sampling methods to detect high-risk HPV for abnormal Pap smear results.

A sample size of 130 women will be sufficient to provide a 10% precision to estimate the kappa coefficient, if the κ is 50% (worst case scenario, as the precision will be better if the κ is lower or higher than 50%). Assuming a 40-50% prevalence of the HPV infection in our selected population, the precision of other measures (sensitivity and specificity) will be more or less 15%.

CONFIDENTIAL

4. Ethical issues

Apart from the collection of a cervical sample by the physician for HVP testing, the colposcopy consultation will not be altered by the study. Any discrepant results between this sample and the self-collected specimen will be discussed with the female patients, verified and managed accordingly.

5. Financial consideration

This study includes no charge for the patients and HUG. Financial support will be solicited for a 20% research nurse for one-year period (CHF 20000), HPV TESTS for all participants (CHF 18600) and other charges (1'400).

6. Expected contribution

We use a cost-effective strategy that if it proves to be as sensitive as the standard HPV testing with v-STM or the actual new alternatives, the FTA cartridge, it will contribute to the development and validation of this method for HPV screening.

CONFIDENTIAL

References

1. *American Cancer Society. Global Cancer Facts & Figures 2nd Edition. .* Atlanta: American Cancer Society, 2011.
2. Mayrand, M.-H., et al., *Human papillomavirus DNA versus Papanicolaou screening tests for cervical cancer.* New England Journal of Medicine, 2007. **357**(16): p. 1579-1588.
3. Ronco, G., et al., *Results at recruitment from a randomized controlled trial comparing human papillomavirus testing alone with conventional cytology as the primary cervical cancer screening test.* Journal of the National Cancer Institute, 2008. **100**(7): p. 492-501.
4. Cox, J.T., *History of the use of HPV testing in cervical screening and in the management of abnormal cervical screening results.* Journal of clinical Virology, 2009. **45**: p. S3-S12.
5. Gök, M., et al., *HPV testing on self collected cervicovaginal lavage specimens as screening method for women who do not attend cervical screening: cohort study.* BMJ: British Medical Journal, 2010. **340**.
6. Nobbenhuis, M.A., et al., *Primary screening for high risk HPV by home obtained cervicovaginal lavage is an alternative screening tool for unscreened women.* Journal of clinical pathology, 2002. **55**(6): p. 435-439.
7. Sherris, J., et al., *Evidence-based, alternative cervical cancer screening approaches in low-resource settings.* International perspectives on sexual and reproductive health, 2009. **35**(3): p. 147-152.
8. Sankaranarayanan, R., et al., *HPV screening for cervical cancer in rural India.* New England Journal of Medicine, 2009. **360**(14): p. 1385-1394.
9. Harper, D.M., et al., *Randomized clinical trial of PCR,Äìdetermined human papillomavirus detection methods: Self-sampling versus clinician-directed,ÄìBiologic concordance and women's preferences.* American journal of obstetrics and gynecology, 2002. **186**(3): p. 365-373.
10. Ogilvie, G., et al., *Diagnostic accuracy of self collected vaginal specimens for human papillomavirus compared to clinician collected human papillomavirus specimens: a meta-analysis.* Sexually transmitted infections, 2005. **81**(3): p. 207-212.
11. Petignat, P., et al., *Are self-collected samples comparable to physician-collected cervical specimens for human papillomavirus DNA testing? A systematic review and meta-analysis.* Gynecologic oncology, 2007. **105**(2): p. 530-535.
12. Dzuba, I.G., et al., *The acceptability of self-collected samples for HPV testing vs. the pap test as alternatives in cervical cancer screening.* Journal of women's health & gender-based medicine, 2002. **11**(3): p. 265-275.
13. Waller, J., et al., *Acceptability of unsupervised HPV self-sampling using written instructions.* Journal of Medical Screening, 2006. **13**(4): p. 208-213.
14. Gustavsson, I., et al., *Use of FTA card for dry collection, transportation and storage of cervical cell specimen to detect high-risk HPV.* Journal of clinical Virology, 2009. **46**(2): p. 112-116.
15. Eperon, I., et al., *Randomized comparison of vaginal self-sampling by standard vs. dry swabs for Human papillomavirus testing.* BMC cancer, 2013. **13**(1): p. 353.
16. Wolfrum, S.G., et al., *Evaluation of dry and wet transport of at-home self-collected vaginal swabs for human papillomavirus testing.* Journal of medical microbiology, 2012. **61**(Pt 11): p. 1538-1545.
17. Geraets, D., et al., *Clinical evaluation of high-risk HPV detection on self-samples using the indicating FTA-elute solid-carrier cartridge.* Journal of clinical Virology, 2013.
18. Guan, Y., et al., *Agreement for HPV genotyping detection between self-collected specimens on a FTA cartridge and clinician-collected specimens.* Journal of virological methods, 2013.
19. Gonzalez, P., et al., *Evaluation of the FTA Carrier Device for Human Papillomavirus Testing in Developing Countries.* Journal of clinical microbiology, 2012. **50**(12): p. 3870-3876.
20. Santos, C.R., et al., *Use of FTA elute card impregnated with cervicovaginal sample directly into the amplification reaction increases the detection of human papillomavirus DNA.* Brazilian Journal of Microbiology, 2012. **43**(1): p. 389-392.
21. De Bie, R.P., et al., *The indicating FTA elute cartridge: a solid sample carrier to detect high-risk HPV and high-grade cervical lesions.* The Journal of Molecular Diagnostics, 2011. **13**(4): p. 371-376.
22. Lenselink, C.H., et al., *Detection and genotyping of human papillomavirus in self-obtained cervicovaginal samples by using the FTA cartridge: new possibilities for cervical cancer screening.* Journal of clinical microbiology, 2009. **47**(8): p. 2564-2570.

CONFIDENTIAL

Annexes

I. Flowchart

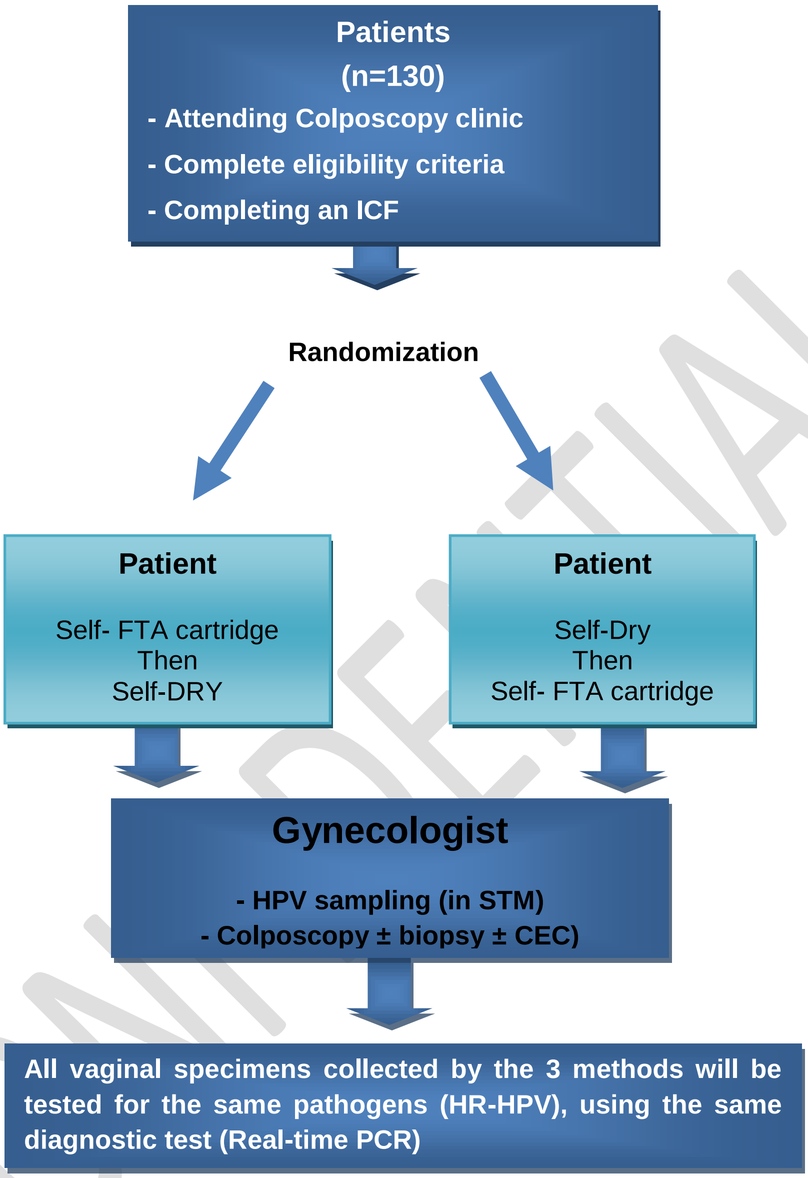

CONFIDENTIAL
